# Analgesic effects of optogenetic inhibition of basolateral amygdala inputs into the prefrontal cortex in nerve injured female mice

**DOI:** 10.1186/s13041-019-0529-1

**Published:** 2019-12-04

**Authors:** Vinicius M. Gadotti, Zizhen Zhang, Junting Huang, Gerald W. Zamponi

**Affiliations:** Department of Physiology and Pharmacology. Hotchkiss Brain Institute, Children’s Hospital Research Institute, Cumming School of Medicine, University of Calgary, Calgary, AB Canada

**Keywords:** Neuropathy, Hyperalgesia, Female mice, Brain circuits

## Abstract

Peripheral nerve injury can lead to remodeling of brain circuits, and this can cause chronification of pain. We have recently reported that male mice subjected to spared injury of the sciatic nerve undergo changes in the function of the medial prefrontal cortex (mPFC) that culminate in reduced output of layer 5 pyramidal cells. More recently, we have shown that this is mediated by alterations in synaptic inputs from the basolateral amygdala (BLA) into GABAergic interneurons in the mPFC. Optogenetic inhibition of these inputs reversed mechanical allodynia and thermal hyperalgesia in male mice. It is known that the processing of pain signals can exhibit marked sex differences. We therefore tested whether the dysregulation of BLA to mPFC signaling is equally altered in female mice. Injection of AAV-Arch3.0 constructs into the BLA followed by implantation of a fiberoptic cannula into the mPFC in sham and SNI operated female mice was carried out, and pain behavioral responses were measured in response to yellow light mediated activation of this inhibitory opsin. Our data reveal that Arch3.0 activation leads to a marked increase in paw withdrawal thresholds and latencies in response to mechanical and thermal stimuli, respectively. However, we did not observe nerve injury-induced changes in mPFC layer 5 pyramidal cell output in female mice. Hence, the observed light-induced analgesic effects may be due to compensation for dysregulated neuronal circuits downstream of the mPFC.

Chronic pain represents a major public health concern and often exhibits a strong affective/motivational component that contributes to the negative impact on the quality of life of affected individuals [1–3]**.** It is known that pain chronification can lead to the remodeling of brain circuits, but the underlying mechanisms remain poorly understood. The medial prefrontal cortex (mPFC) is known to be involved in the processing of neuropathic pain and is also important for emotional aspects of pain [4, 5]**.** We have previously shown that mice subjected to spared injury of the sciatic nerve (SNI) undergo remodeling of the prelimbic area of the mPFC as a result of enhanced feed-forward inhibiton of layer 5 pyramidal cells by parvalbumin expressing GABAergic interneurons [6]. Optogenetic activation of layer 5 mPFC pyramidal cells reversed mechanical and thermal hypersensitivity in SNI mice [6] indicating that these circuit changes are causal. The mPFC receives neuronal projections from the basolateral amygdala (BLA) [7] which may contribute to affective-motivational aspect of neuropathic pain states [1, 8]. We recently identified a BLA - mPFC – periaqueductal gray (PAG) – spinal cord (SC) pathway that links injury-induced alterations of BLA to mPFC inputs to a loss of descending modulation of pain signals in the SC [9]. Glutamatergic inputs from the BLA onto GABAergic inhibitory interneurons located in mPFC were found to be increased following SNI, as a result of weakened endocannabinoid inhibition of these inputs [9]. Through the application of combined optogenetic and pharmacological approaches we were able to manipulate this BLA-PFC-PAG-SC circuitry and reverse both mechanical and thermal hypersensitivity in male SNI mice at various loci within this circuit [9]. Importantly, Arch3.0-mediated inhibition of glutamatergic BLA to mPFC projections reversed tactile allodynia, cold allodynia and thermal hyperalgesia, along with observed alterations in place escape/avoidance in male SNI mice [9].

The experiments described in our previous studies were conducted exclusively in male mice. There is evidence that some signalling processes involved in chronic pain states exhibits marked sex differences [10–12]**.** It is thus crucial to examine whether inhibiting glutamatergic BLA to mPFC inputs also produces analgesia in female neuropathic mice**.** Hence, 7 week old female C57Bl/6 J mice purchased from Jackson Laboratories received AAV5-CamKIIα-Arch3-eYFP injections into the BLA (500-600 nL, obtained from the University of North Carolina Vector Core (Chapel Hill, NC), at 4 × 10^12^ viral genomes per milliliter) to induce the expression of Arch3.0. Two weeks later, a fiberoptic cannula (2.5 mm ceramic ferrule, 2.0 mm length, Thorlabs) was implanted into the mPFC so that the inhibitory opsin could be activated by yellow light stimulation. This approach allows yellow light-mediated inhibition of BLA inputs into the mPFC via acivation of Arch3.0 expressed in the nerve terminals. After a further two week period, a baseline sensory measurement was conducted and then SNI surgeries encompassing a ligation and transection of the peroneal and tibial nerves with a 6–0 silk suture (Ethicon, USA) were performed. Animals that did not display neuropathic pain responses (i.e., no decrease in mechanichal threshold or thermal latency) after 14 days were discarded. Sensory pain analysis was carried out between 7 and 8 weeks after AAV injections, and thus between 2 and 3 weeks following SNI surgery. On the testing day, prior to experimentation, animals were allowed to habituate for at least 90 min inside individual plexiglass chambers on top of a grid floor, with the implanted brain cannula connected to a DPSS laser (yellow 589 nm, Laserglow Technologies, Ontario). Before light stimulation (Light OFF) their ipsilateral (nerve-injured side) and contralateral hindpaws were first assessed for mechanical withdrawal threshold and thermal withdrawal latency, using respectively a Dynamic Plantar Aesthesiometer and a Hargreaves Apparatus (both from Ugo Basile, Varese, Italy). Each hindpaw was measured 3 times. After 30 min, animals received continuous delivery of yellow light (Light ON) beginning 3 min before measurements of mechanical thresholds and thermal withdrawal latencies. Experimental protocols were performed exactly as described in our recent work [9] and all experimental procedures were approved by the Animal care committee of the University of Calgary.

Fig. 1a shows that similarly to what we had observed in male mice, yellow light-mediated activation of Arch3.0 (20 mW at the fiber tip, S130C power sensor, Thorlabs) significantly inhibited mechanical hyperalgesia in female mice with differences revealed by two-way ANOVA between the ipsilateral and contralateral paw interaction (*p* < 0.0004, F = 2.698 (4,4) and for the before vs after light stimulation interaction (*p* < 0.0058, F = 2.074 (4,4). Optogenetic inhibition of BLA inputs also significantly decreased thermal hyperalgesia **(**Fig. 1b**)** (two-way ANOVA between ipsilateral and contralateral paw (*p* < 0.0001, F = 3.532 (4,4) and light ON vs light OFF interaction (p < 0.0001, F = 1.776 (4,4)). There was no effect of Arch3.0 activation on sensory responses on the contralateral side. A detailed view at our present and prior results shows that female animals exhibited somewhat augmented injury-induced thermal hyperalgesia compared to males, and a stronger analgesic response to thermal stimuli upon activation of Arch3.0 [9]. Whether these differences reside in differences in the primary afferent pathway or in slightly different processing within the brain is unclear. Nonetheless, these data indicate that inhibition of glutamatergic inputs into the mPFC, and thus GABAergic feed-forward inhibition in this region, produces analgesia in both male and female mice.

**Fig. 1:**
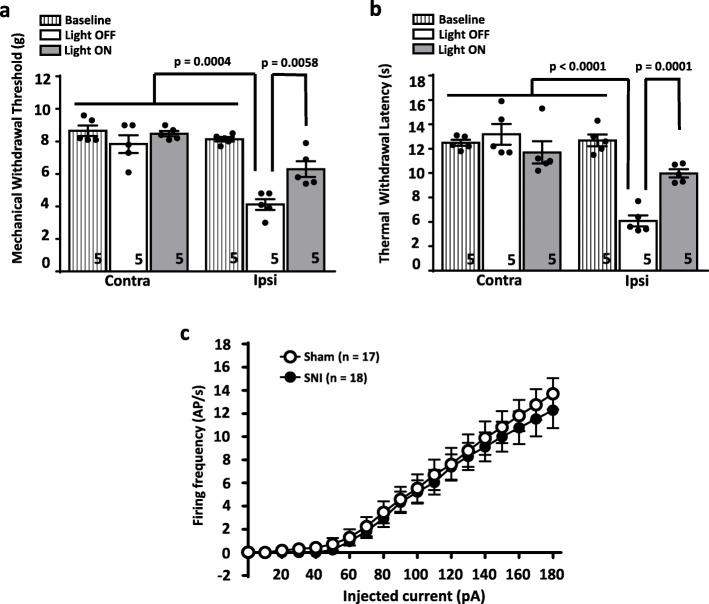
Effect of optogenetic manipulation of BLA inputs into the prelimbic mPFC of female neuropathic mice. **(a)** Mechanical paw withdrawal threshold and **(b)** thermal paw withdrawal latency in the ipsilateral hindpaws before nerve injury (baseline), and after SNI with (light ON) and without (light OFF) activation of Arch3.0 expressed in the BLA to prelimbic mPFC projection. Data were analyzed with Graph Pad Instat 3.0 and Graphpad Prism 6.0 and are presented as mean ± SEM with two way analysis of variance (ANOVA) followed by Tukey post hoc corrections. Statistical significance was accepted at the level of *p* < 0.05.Numbers shown in the bars reflect numbers of mice. **(c)** Current clamp recordings from putative large triangular layer 5 pyramidal cells in mPFC slices from sham (17 cells from 4 animals) and SNI (18 cells from 3 animals) mice. Action potential frequencies are shown in response to different levels of depolarizing current injections. The data sets are not statistically different from each other. Membrane potentials were held at − 70 mV by injecting a small bias current (Resting membrane potentials are similar in Sham: − 68.56+/− 0.96 mV and SNI: 69.05+/− 0.80 mV; *p* = 0.6981, unpaired t-test). To assess general cell excitability of pyramidal cells, no synaptic blockers were added in perfusion solutions (For detailed methods, see Ref [6])

Layer 5 pyramidal neurons integrate monosynaptic excitatory inputs from the BLA, and di-synaptic GABAergic feed forward inhibition mediated by glutamatergic BLA inputs into GABAergic interneurons in the mPFC. In male mice, this causes an overall reduction in pyramidal cell activity [6, 9]. Interestingly, when we examined the firing properties of layer 5 pyramidal cells using current clamp recordings in mPFC slices from female C57 mice (using analagous electrophysiological approaches as those described by us previously for male animals [6]), a surprising result was obtained. As shown in Fig. 1c, two weeks after SNI surgery, pyramidal cell firing frequency was indistinguishable between female sham operated and SNI mice. These data may indicate that there is either no injury-induced dysregulation of synaptic inputs into the mPFC, or that the integration of such inputs via the GABAergic interneuron circuitry within the mPFC fails to manifest itself as a reduction in layer 5 output. How can these data be reconciled with the observations in Fig. 1a and b? It is possible that peripheral nerve injury in female mice may lead to dysregulation of brain structures downstream of the mPFC, such as the PAG region or its ensuing descending projections. If so, then boosting mPFC output by optogenetically inhibiting feed forward inhibition mediated by BLA inputs could serve as a beneficial compensatory mechanism. Further experimentation will be necessary to test such a hypothesis.

Chronic neuropathic pain can be a debilitating condition and is often refractory to traditional pharmacological strategies. Recent evidence suggests that there are important sex differences in the processing and maintenance of chronic neuropathic pain states [10–12] and this has to be considered when aiming towards the development of novel and efficacious therapeutic strategies. Our findings here reveal that there are indeed important sex differences with regard to the nerve injury-induced dysregulation of mPFC function, but that the mPFC may be a brain cicuit that could be targeted equally effectively in both sexes for tackling chronic neuropathic pain.

## Data Availability

The data used in our study are available from the authors on reasonable request.

## Abbreviations

AAV: Adeno-associated virus
Arch 3: Archaerhodopsin 3.0
BLA: Basolateral amygdala
mPFC: Medial prefrontal cortex
PAG: Periaqueductal grey
PFC: Prefrontal cortex
SC: Spinal cord
SNI: Spared nerve injury
